# The Impact of Shiftwork on Skeletal Muscle Health

**DOI:** 10.3390/nu9030248

**Published:** 2017-03-08

**Authors:** Brad Aisbett, Dominique Condo, Evelyn Zacharewicz, Séverine Lamon

**Affiliations:** 1 School of Exercise and Nutrition Sciences, Deakin University, Geelong 3220, Australia; dominique.condo@deakin.edu.au (D.C.); e.zacharewicz@deakin.edu.au (E.Z.); severine.lamon@deakin.edu.au (S.L.); 2 Institute for Physical Activity and Nutrition (I-PAN), Deakin University, Geelong 3220, Australia

**Keywords:** protein intake, resistance training, sleep, hormones

## Abstract

(1) Background: About one in four workers undertake shift rosters that fall outside the traditional 7 a.m.–6 p.m. scheduling. Shiftwork alters workers’ exposure to natural and artificial light, sleep patterns, and feeding patterns. When compared to the rest of the working population, shiftworkers are at a greater risk of developing metabolic impairments over time. One fundamental component of metabolic health is skeletal muscle, the largest organ in the body. However, cause-and-effect relationships between shiftwork and skeletal muscle health have not been established; (2) Methods: A critical review of the literature was completed using online databases and reference lists; (3) Results: We propose a conceptual model drawing relationships between typical shiftwork consequences; altered light exposure, sleep patterns, and food and beverage consumption, and drivers of skeletal muscle health—protein intake, resistance training, and hormone release. At present, there is no study investigating the direct effect of shiftwork on skeletal muscle health. Instead, research findings showing that acute consequences of shiftwork negatively influence skeletal muscle homeostasis support the validity of our model; (4) Conclusion: Further research is required to test the potential relationships identified in our review, particularly in shiftwork populations. Part of this testing could include skeletal muscle specific interventions such as targeted protein intake and/or resistance-training.

## 1. Introduction

In North America, continental Europe, and Australia, more than 15% of the workforce undertake shifts that include work hours outside 7 a.m. to 6 p.m. [1]. These workers deliver essential 24-h services for communities, including healthcare, construction, and emergency response [2,3,4,5]. To maintain these 24-h services, shiftworkers endure continual acute and chronic risks to their health and safety [6]. In the short-term, sleep deprivation and disturbances lead to impaired decision making and vigilance on the job, which can increase the risk of workplace accidents [7]. Disruptions to sleep and waking habits can also adversely influence social and family relationships [8], which can diminish job retention [9] and quality of life. Shiftworkers frequently exposed to irregular working hours also face significantly greater risks of developing diabetes [10], obesity [11], and cancer [12]. 

Together with the short- and long-term health and safety risks they face, the essential role that shiftworking populations serve in their communities makes them a high priority research area. To this end, considerable research has already focused on shiftworkers’ sleep impairments [6], and associated risk of short-term injuries [7] and longer-term metabolic diseases [10,11,13,14]. One area that has received relatively little attention, but is pertinent to both short-term work capacity and long-term metabolic health is the impact of shiftwork on skeletal muscle health. Skeletal muscle is a major metabolic tissue that is not only essential for all human movement, but also constitutes a critical storage organ for essential substrates and plays a major role in energy production and metabolism [15,16]. Maintaining skeletal muscle health over a lifespan underpins physical work capacity [17] and is particularly relevant for many shiftworking populations who must perform physically demanding tasks and/or maintain standing or upright postures across shifts lasting eight or more hours [18]. Disruption in muscle homeostasis is associated with metabolic and chronic diseases that are overrepresented in shiftworking populations [10,11,13,14]; however, no cause-and-effect relationship has been established between shiftwork and poor skeletal muscle health. 

**Figure 1:**
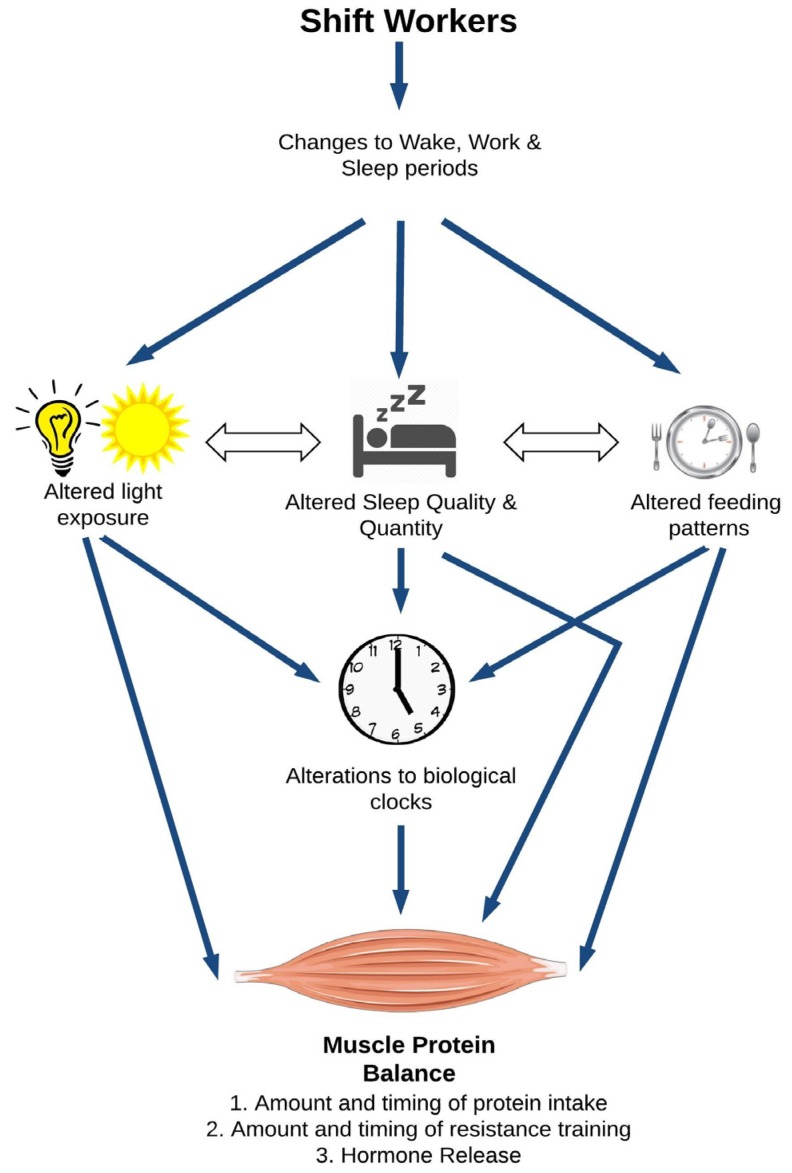
A model for the potential impact of shiftwork on skeletal muscle health.

The review will begin by defining shiftwork schedules and typical shiftwork industries. Thereafter, we will provide an overview of skeletal muscle physiology, and briefly describe the major drivers of skeletal muscle health. We will then propose a conceptual framework for the direct and indirect pathways through which shiftwork could impair skeletal muscle health (Figure 1). The bulk of the review will draw on available evidence supporting our hypothesis that shiftwork and its acute consequences impair skeletal muscle health. These sections will focus on the direct impacts of circadian disruption on skeletal muscle regulation as well as the impact of shiftwork and sleep disruption on food and beverage choices, and hormonal changes. The review will conclude with nutrition- and exercise-based targeted intervention areas that interested research groups may trial as part of a new research agenda for shiftworkers’ health.

### 1.1. Shiftwork

For the purposes of this review, shiftwork will comprise working hours that exist outside of the traditional 7 a.m. to 6 p.m. scheduling [1]. The various types of shiftwork patterns have been recently described in seminal reviews by Wright et al. [1] and Kecklund and Axelsson [6]. Briefly, night-shift hours typically range from 9 p.m. to 8 a.m.; evening shifts from 2 p.m. to 12 a.m.; and early morning shifts include any work starting between 4 and 7 a.m. [1]. Early morning and night shifts acutely lead to shorter sleep periods, whilst evening shiftwork is associated with the longest sleep durations [6]. Such rosters are typical of nursing, medicine and paramedicine, maintenance and factory work, aviation, and emergency services [1,5,6,18]. Many sectors use a rotating shift system where workers will rotate through a period of day to evening (where applicable) to night shift. The number of consecutive shifts of each type impacts sleep, with longer rotations (i.e., four to seven consecutive shifts of the same type) leading to ~25 min more sleep than faster rotations [6]. Changing from a day to night shift can lead to a period of total sleep deprivation, particularly on the first night shift [19]. The exposure to total sleep deprivation is also typical for emergency service workers who may be called to an incident (e.g., road crash, storm, or wildfire) in the afternoon or early evening after working all day [2,3,4]. In some emergency service jurisdictions, workers are able to rest or sleep at the station or in their homes until an emergency response is required [5]. In these environments, workers face disrupted sleep and partial sleep restriction that can evoke different physiological responses when compared to total sleep deprivation [5]. In this review, we will strive to draw on studies using actual or simulated shiftwork schedules to understand their potential impacts on skeletal muscle health. As disrupted sleep is a typical consequence of many schedules, we will also draw on experimental evidence using complete sleep deprivation and partial sleep restriction models. Special care will be taken to alert the reader of the potential impact that different types of shiftwork could have on skeletal muscle health in both acute and chronic settings.

### 1.2. Skeletal Muscle Health

A fundamental component of human metabolic health, physical work capacity, and quality of life is skeletal muscle health [17]. Skeletal muscle is a very plastic tissue able to rapidly modify its structure, function, and metabolism in response to internal and external stress signals. Comprising 40% of the total body mass [17], skeletal muscle is the largest human organ. It primarily functions as a structural support unit, enabling the body to maintain posture and perform gross and fine motor movements [20]. Skeletal muscle is composed of a heterogeneous collection of muscle fibres. Each fibre is a multinucleated muscle cell constituted of myofibrils, the proteinic structure made of actin and myosin that allows for muscle contraction. The different types of muscle fibres allow for the wide variety of capabilities of this organ; with fast-twitch fibres (type II) producing a contractile response that is faster but subjected to higher fatigability than slow twitch fibres (type I). Skeletal muscle is also a critical storage organ for essential substrates and plays a major role in energy production and metabolism [15,16]. Numerous substrates including muscle glycogen, blood glucose, and free fatty acids derived from muscle or adipose tissue can be used to generate ATP, the main source of energy that sustains muscle contraction [21]. Disruption in muscle energy metabolism is associated to metabolic disorders, including obesity and diabetes, and neurodegenerative conditions such as motor neuron disease. Of particular interest for this review, skeletal muscle houses 50%–75% of the body’s total protein pool [17]. Skeletal muscle proteins are constantly built up (protein synthesis) and broken down (protein degradation) [17]. In normal physiological conditions, the amount of proteins synthetized in the muscle balances the amount of proteins degraded in the muscle, resulting in steady muscle mass [22]. Protein degradation can exceed protein synthesis during periods of serious disease, immobilization, and aging [23], leading to a net loss of muscle mass. In addition, muscle protein metabolism is disrupted during periods of malnutrition and starvation, where the muscle becomes a vital substitute fuel supply for the brain and immune system [17]. Compromising muscle protein metabolism results in a net loss of muscle mass [24,25,26] and prevents optimal muscle function. Low muscle mass is a hallmark of numerous metabolic challenges. These include chronic and metabolic disorders, such as cancer, AIDS, and neuromuscular conditions [23]. Maintaining the balance between protein synthesis and protein degradation over a lifespan is therefore critical for physical performance, metabolic health, and general wellbeing [17]. The muscle protein metabolism balance is tightly regulated by a combination of anabolic (pro-protein synthesis) and catabolic (pro-protein degradation) stimuli including (1) the ingestion of dietary protein (anabolic); (2) resistance exercise (anabolic) and (3) circulating signaling molecules, including metabolic hormones and myokines (anabolic or catabolic) [17].

## 2. A Model for Shiftwork and Skeletal Muscle Health

The overarching hypothesis of this review is that shiftwork may significantly impair skeletal muscle health through multiple physiological pathways, resulting in a reduction of protein synthesis and an augmentation of protein degradation in the muscle (Figure 1). At the top of Figure 1, shiftwork alters the sleep-wake cycle of the worker. In this review, we will focus on three typical consequences of the altered sleep-wake cycle, namely altered light exposure, sleep, and feeding patterns. Altered light exposure refers to the reduction in natural night and increase in artificial light, particularly at night. Shiftworkers’ sleep patterns are characterized by a reduced quality and quantity of sleep and, depending on their schedule, a shift in their main sleep including (for some) sleeping primarily during day time hours. Similarly, shiftworkers’ eating patterns move with their altered sleep-wake cycle, but shiftworkers also make different food and beverage choices than daytime workers. There are also several interactions between these sub-categories. Individually and collectively, changes in light, sleep, and feeding patterns can all influence the body’s circadian clocks depicted in the middle of Figure 1. This image refers to the suprachiasmatic nucleus (SCN), the central clock of the body that governs tissue homeostasis. In addition, skeletal muscle possesses its own intrinsic biological clock, which is also sensitive to the changes in light exposure, wake, sleep, and dietary patterns experienced by shiftworkers [27]. The regulation of muscle homeostasis is multi-factorial, with protein intake, resistance training, and hormonal patterns independently influencing muscle protein balance. Beyond impacts on the central and peripheral circadian clocks, alterations to light, sleep, and feeding patterns may also directly impact muscle protein balance. Here we will primarily focus on the studies reporting muscle data. Human experimental data will be prioritized and supplemented, where appropriate, with evidence from animal studies. While this approach limits the number of studies discussed in our review, our intent is to stay focused on new contributions to the already large body of research addressing shiftworkers’ health.

### 2.1. Direct Effects of Circadian Disruption on Skeletal Muscle Tissue

Skeletal muscle fibres possess their own intrinsic biological clock, which relies on a multi-level transcriptional and translational feedback loop system involving the core clock genes *Clock*, *Bmal1*, *Cry1*/*2*, and *Per 1*/*2* (reviewed in [28,29]). The muscle biological clock is primarily studied in rodent models. While the obvious limitations to this approach should be kept in mind, novel data are often only available from mouse and rat models. In mice, seventeen percent of all genes display circadian-like regulation patterns in skeletal muscle [30], including *Myod1*, a muscle-specific transcription factor playing a key role in muscle development [31]. These genes are involved in all aspects of muscle biology, including growth, function, and metabolism [28,29,32], where nearly 30% of all genes directly regulated by *Clock* are involved in energy metabolism [30]. It follows that disruptions in the circadian clock are strongly associated to skeletal muscle health. For example, a functional biological clock is essential to the normal secretion of basal myokines in vitro [33] and to the maintenance of muscle structure and function in rodents [27,31]. A series of recent animal studies have linked the specific loss-of-function of one of the core clock genes to a range of muscle phenotypes, including muscle atrophy, structural impairments, altered metabolism, altered regeneration, and reduction in force and endurance (reviewed in [32]). 

Few human studies have investigated the direct relationships between the acute impacts of shiftwork (e.g., altered light exposure, sleep, and feeding patterns; Figure 1) and skeletal muscle health. One night of complete sleep deprivation decreased the mRNA levels of the clock genes *Bmal1* and *Cry1* in 15 healthy men [34]; however, whether light exposure during the trial or sleep deprivation itself drove these changes is unknown. The possible interplay between sleep restriction, diet, and skeletal muscle health has been evidenced in less controlled field studies. Nindl et al. [35] showed that soldiers who were only permitted 1–3 h sleep per night during a physically intense four-day training period lost 3% fat free mass. These soldiers were also subjected to severe caloric restriction each day. Unpacking the relative contribution of the sleep and calorie restriction was not possible. However, a more recent study showed that sleep loss could moderate the relative impact of calorie restriction on skeletal muscle health [36]. Nedeltcheva et al. [36] showed that participants following a 14-day calorie restricted diet lost 60% more muscle mass when sleep restricted (5.5 h sleep per night) than those who slept normally (8.5 h sleep per night).

In the relative absence of human data, the proposed association between the acute impacts of shiftwork and skeletal muscle health can be supported by rodent studies. In rats submitted to 96 h of rapid eye movement (REM) sleep deprivation, there was a significant decrease in tiblias anterior muscle mass [37], gastrocnemius mass/tibia length ratio [38], average fibre cross-sectional area (CSA, a direct measure of muscle size) [37], and type II (fast-twitch) fibre CSA [38]. The phosphorylation levels of the molecular markers of muscle protein synthesis were reduced, indicating a reduction in muscle protein synthesis with sleep deprivation. Conversely, the activity of the ubiquitin proteasome system increased, indicating greater protein degradation [38]. Sleep-deprivation induced muscle atrophy could be partially restored by a 96-h recovery period [37]. Work from the same group confirmed that a decrease in average muscle fibre CSA induced by sleep-deprivation was specific to glycolytic and mixed muscles and that 96 h of paradoxical sleep deprivation significantly reduced fat deposition in all types of muscle, possibly as a direct consequence of a negative energy balance [39]. Rats subjected to sleep deprivation for 18 h per day demonstrated an increase in the *Mhc1* gene and protein expression (a protein that is characteristic of slow-twitch fibres) and a decrease in the *Mhc2* gene and protein expression (a protein that is characteristic of fast-twitch fibres) in the masseter muscle after 7 and 14 days; values that returned to baseline levels after 21 days of sleep deprivation [40]. In contrast, rats subjected to 3–14 days of sleep deprivation did not display an increase in oxidative stress in skeletal muscle [41]. More recently, a mouse study using continuous light exposure confirmed that a functional circadian system is required to maintain skeletal muscle health. A six-month period of light exposure induced a number of muscle dysfunctions, including reduced grip strength and grip hanging duration; impairments that were mostly reversed when the animals were returned to a normal day/night cycle. It was proposed that these changes were mediated by changes in SCN neural activity [42]. More work is required to reconcile the acute and sustained impacts that sleep deprivation and/or changes in light exposure has on skeletal muscle, particularly in humans.

### 2.2. Shiftwork, Sleep Disruption, and Food and Beverage Choices

**Figure 2:**
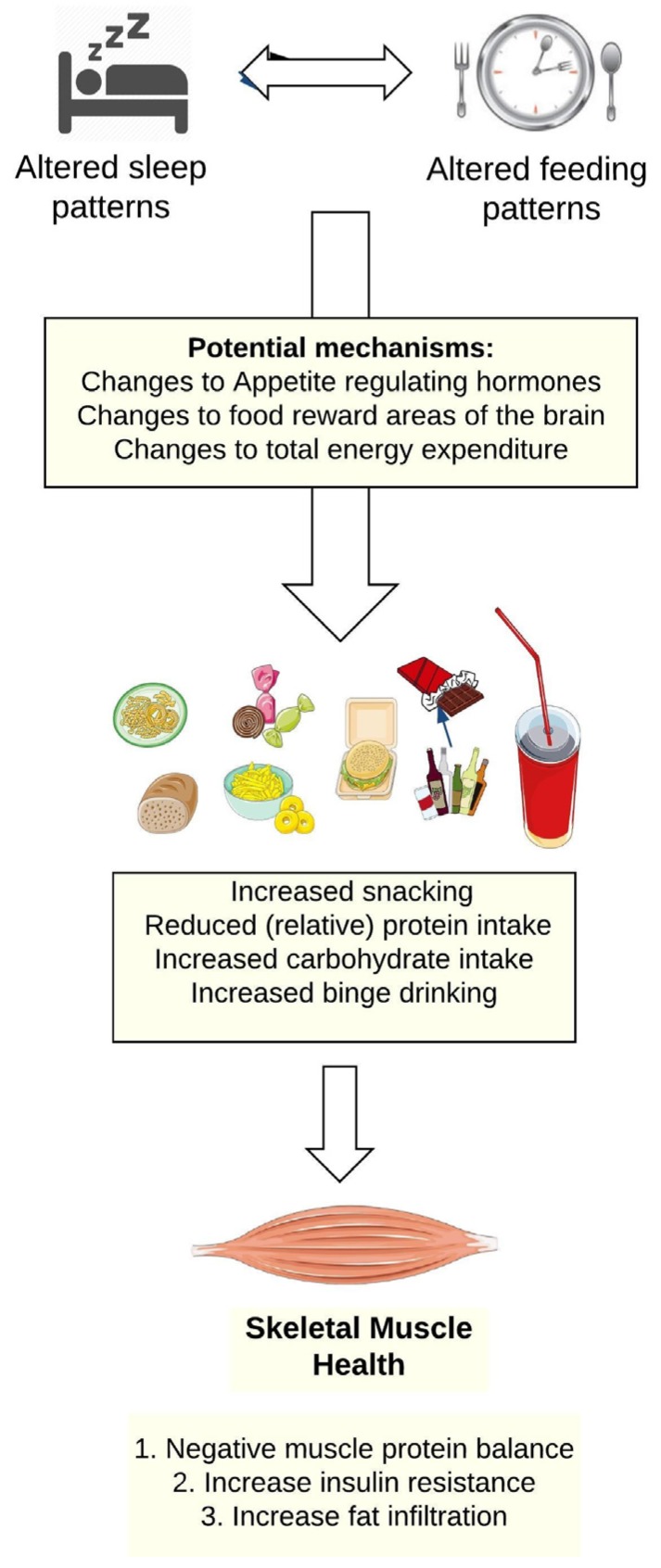
A model of how the altered sleep and feeding patterns of shiftworkers could impair skeletal muscle health.

As presented in Figure 1, shiftwork alters workers’ sleep-wake patterns, which in turns leads to changes in their food and beverage consumption. Figure 2 focuses on the potential pathways through which changes in food and beverage intake could impair skeletal muscle health. 

Protein ingestion is one of the three primary drivers of protein synthesis in skeletal muscle [17]. As muscle tissue houses 50%–75% of the body’s total protein pool [17], dietary protein supplies the materials needed to replenish and remodel muscle cells by increasing systemic amino acid availability and improving insulin sensitivity [43]. The type, amount, and timing of protein ingestion can be modulated to increase protein synthesis rates and improve net protein balance within skeletal muscle [44]. Twenty to 30 g of high quality protein containing approximately 10 g of essential amino acids is required to maximally stimulate maximal protein synthesis [45,46]. Protein intakes of 0–10 g produced significantly lower protein synthesis, with no additional benefit observed when >30 g was ingested [45,46]. Moderate amounts of protein spread evenly throughout the day causes a 25% increase in muscle protein synthesis compared to a skewed intake towards the end of the day. Therefore, low or irregular protein intake can result in a negative protein balance, with protein degradation surpassing protein synthesis and promoting net muscle loss. The amino acid profile and rate of digestibility can also influence protein synthesis. Protein sources consisting of essential amino acids doubled protein synthesis rates compared to equal amounts of non-essential amino acids [47]. Leucine, a branched chain amino acid, is particularly efficient as it stimulates muscle protein synthesis both directly and indirectly through the secretion of insulin from the pancreatic β cells [48]. Finally, rapidly digestible proteins such as whey result in a greater stimulation of post prandial muscle protein synthesis when compared to slowly digestible proteins such as casein [49].

The diets of shiftworkers have been reviewed by Lowden et al. [50]. These authors did not specifically focus on protein intake in shiftworkers, though some evidence suggested high ingestion of animal protein and fats by nightshift workers. A more consistent pattern is that despite little difference in energy intake between different work groups, fat and refined carbohydrates comprise a greater portion of shiftworkers’ diets compared to daytime workers. Such dietary patterns can increase the risk of insulin resistance, promoting muscle protein degradation, favoring fat infiltration in the muscle and negatively impacting skeletal muscle health (Figure 2). 

As shiftwork disrupts workers’ sleep patterns, studies documenting food choices following sleep deprivation and sleep restriction may provide further insight into the links between shiftwork, diet, and skeletal muscle health. Sleep loss generally increases the preference for foods with a high carbohydrate content including sweets, salty, and starchy foods compared to vegetables, fruit, and high protein foods [51,52,53,54]. Across two weeks of sleep restriction (i.e., 5 h sleep per night) [55] or following one night of total sleep deprivation [56], participants increased their ad-libitum food and energy intake, particularly through increased snacking. Sleep restriction also impacts food-purchasing behaviours, influencing longer-term food intake. After one night of total sleep deprivation and following a calorie controlled breakfast, one study reported an increase in the purchase of higher calorie foods, including sweet and fatty food items, and total grams of food within the same budget compared to a night of sleep [57]. Neurological factors may contribute to explain these results, with imaging studies showing a greater response in the food reward areas of the brain to unhealthy food items compared to healthy food items following sleep restriction [58,59].

The mechanisms by which shiftwork may alter dietary choices could also include alterations in appetite regulating hormones, leptin (appetite-inhibiting hormone), and ghrelin (appetite-stimulating hormone [60]); however, direct evidence is lacking. Men working dayshifts recorded lower concentrations of leptin compared to other shifts [61]. However, early morning shiftworkers had lower ghrelin concentrations over the day, consumed less total energy, and reported lower appetite ratings when compared to day and night shiftworkers [61]. These potentially conflicting findings are further confounded by the direct impact of sleep restriction (common to many shiftworkers) on the same hormones. For instance, a randomised crossover study found reduced leptin and increased ghrelin following two consecutive nights of sleep restriction (4 h in bed) when compared to sleep extension (10 h in bed), under strict calorie control in the form of intravenous glucose [52]. It follows that hunger and appetite were increased following sleep restriction and were proportional to the increased ghrelin to leptin ratio [52]. Other studies have reported inconsistent results, with either increases in leptin following a sleep restriction period [55,62], no change in satiety [62], or no difference in leptin or ghrelin levels following sleep restriction, despite an increased calorie intake [51]. Teasing out the relationships between the different types of shiftwork, changes to appetite hormones, and food reward centers in the brain should be a focus of future research. Only then can the precise mechanisms for the changes in dietary patterns observed in shiftworkers be understood and targeted interventions be designed. 

Shiftworking also influences alcohol consumption [63,64]. Dorrian and colleagues [63,64] reported that whilst shiftworkers do not drink more than those on standard work rosters, they are at increased risk of ‘binge’ drinking; that is, periods of heavy drinking followed by abstinence. Workers on 12-h rotating shifts consumed more drinks in a single 24-h period compared to those on 8-h rotating shifts [63]. These findings also align with a recent systematic review demonstrating a positive relationship between long working hours and alcohol consumption [65]. For the current review, an increased risk of binge drinking, perhaps during ‘down periods’ between shift ‘blocks’, could pose significant risks to skeletal muscle health. Recently, Parr and colleagues [66] have shown that heavy alcohol consumption reduces muscle protein synthesis rates and increases protein degradation [67] following a bout of combined resistance and endurance exercise. These participants were however well-rested, night-time sleepers so it is unclear whether the observed impairments of alcohol on skeletal muscle growth could be further exaggerated by the changes in sleep patterns (incl. sleep restriction) encountered by shiftworkers.

### 2.3. Shiftwork, Sleep Disruption, and Hormonal Changes

Hormones are circulating molecules that transmit physiological signals to organs, including to skeletal muscle. By binding to specific receptors expressed at the surface of the muscle fibre, hormones trigger the activation of molecular transduction pathways that regulate cell metabolism, structure, and function. In this section, we will draw on the small group of studies describing the hormonal profile exhibited by shiftworking populations. This pool of research will be supplemented by experimental evidence demonstrating the direct impact of sleep deprivation or restriction on the major anabolic and catabolic hormones, including testosterone, insulin and insulin like growth factor 1 (IGF-1), and cortisol. 

#### 2.3.1. Testosterone

Testosterone is a key regulator of skeletal muscle mass that directly promotes muscle protein synthesis [68] while also repressing the negative effect of genes activating protein breakdown [69]. Plasma testosterone levels rise with the onset of sleep and peak after the first REM sleep opportunity. At least 3 h of normal sleep are required to induce this rise [70]. Concentrations remain high until waking and then decrease gradually during the day. Early, underpowered field studies investigating the secretory pattern of testosterone in shiftworkers during and following their night shift reported decreased nocturnal [71] and diurnal [72] testosterone concentrations in serum. More recently, Jensen et al. [73] reported that police officers completing 2, 4, and 7 consecutive nights of night shift (coupled with equivalent recovery days) did not experience any change to their testosterone profiles. However, the authors did not report whether total testosterone (e.g., area under the curve) was affected. A study comparing sex hormone profiles in a heterogeneous population of night-versus day workers revealed that night workers had higher levels of androgen hormones, including testosterone, than day workers. However, this trend was not apparent when looking at males only. The testosterone peak was significantly delayed in night workers, independently of sex [74]. 

The direct relationship between sleep and testosterone in experimental settings has received more attention. Total sleep deprivation, sleep restriction, and fragmented sleep over a 24-h period, which could relate to the work of emergency response or on-call workers [2,3,4,5], respectively lower testosterone levels [71,72] and delay the normal nocturnal blood testosterone rise [75]. The nocturnal rise in testosterone appears dependent on REM sleep with the amplitude of this rise being significantly lower in participants who did not have any REM sleep opportunity [75]. Sustained sleep restriction, which is also representative of some shiftworking conditions, impairs testosterone release as well. After five nights of sleep restriction (4 h sleep per night), there was a trend (*p* = 0.09) for a reduction in total daytime testosterone [76]. Failure to reach statistical significance may reflect that the study was slightly underpowered, the period of sleep restriction was not long enough, or that the total testosterone measure was not sufficiently sensitive to pick up diurnal patterns. Indeed, eight nights of sleep restriction (5 h sleep per night) led to significantly decreased testosterone levels during waking hours, with differences being especially apparent between 2 p.m. and 10 p.m. [77]. Finally, a study compared the effects of shifting sleep (day sleep) and control conditions (night sleep) on circulating testosterone levels. Testosterone concentrations generally raised during sleep and dropped during waking, with small circadian effects being reported [78]. On balance, it appears that shiftwork, and total and sustained sleep restriction can all impair testosterone release, which may fail to promote muscle protein synthesis and preserve skeletal muscle health in affected populations.

#### 2.3.2. Insulin and Insulin-Like Growth Factor 1 (IGF-1)

Insulin is a central regulator of skeletal muscle health and metabolism. This peptidic hormone concurrently increases the postprandial transport and delivery of amino acids to skeletal muscle, activates muscle protein synthesis, and inhibits muscle protein degradation [79]. Maintaining optimal insulin function is essential for metabolic health. Insulin resistance, a metabolic disorder that is characteristic of diabetes and obesity, blunts muscle protein synthesis and promotes muscle protein degradation. By favouring fatty acid uptake in the muscle, insulin resistance also augments fat infiltration into the muscle, leading to a negative muscle protein balance and decreased muscle mass [80,81]. 

The adverse impact of shiftwork and sleep restriction on insulin resistance is suggested through the consistent associations existing between shiftwork, sleep restriction, diabetes, and obesity [6]. Drawing on evidence from 38 meta-analyses and 24 systematic reviews, Kecklund and Axelsson [6] reported that diabetes (relative risk (RR) ratio: 1.09 (95% CI: 1.05 to 1.12)) was more prevalent in shiftworking than in control populations. While no data focused on shiftwork per se, sleep restriction studies returned a RR of 1.25 (95% CI: 1.14 to 1.3) for obesity/weight gain. In experimental settings, Buxton et al. [82] demonstrated reduced insulin sensitivity following as little as one week of sleep restriction (5 h per night) in 20 healthy men. Supporting this finding, five nights of sleep restriction (4 h per night) led to reduced whole body and peripheral insulin sensitivity that was associated with an increase in fasting non-esterified fatty acid in 14 healthy participants [83]. 

IGF-1 is another positive regulator of muscle protein synthesis that is the main activator of the Akt/mTOR pathway in muscle, while also carrying out signaling in other anabolic pathways [84]. Controlled human studies investigating the direct effects of sleep deprivation or restriction on IGF-1 levels are lacking; however, 25 h of sleep deprivation induced significant decreases in free IGF-1 concentrations, while one night of recovery efficiently restored basal circulating IGF-1 levels [85]. Whether chronic changes in IGF-1 concentrations are associated with shiftwork schedules still needs to be investigated. 

#### 2.3.3. Cortisol

Cortisol is the major hormone released through activation of the hypothalamic-pituitary-adrenal axis in response to psychological or physiological stressors [86] in humans. Elevated cortisol levels upset skeletal muscle protein balance by suppressing protein synthesis and promoting protein breakdown [84]. Cortisol inhibits IGF-1 production in skeletal muscle, while up regulating key inhibitors of protein synthesis [84]. Furthermore, cortisol stimulates the major protein degradation pathways, including the ubiquitin–proteasome system [84]. Under normal conditions, cortisol release follows a diurnal pattern characterized by a peak upon morning wakening, followed by a decline across the day, reaching its lowest level ~3–5 h after night-time sleep onset [86]. Across a variety of occupations and work rosters, shiftwork disrupts this diurnal pattern. Studies in police, nursing, and other emergency services demonstrated cortisol dysregulation after short- and longer-term exposure to shift- and, in particular, night-work rosters [87,88,89]. The impairment is typically characterized as suppressed cortisol awakening response, followed by a slower rate of decline, which can lead to an elevated night-time cortisol levels [89,90]. Depending on the number of samples collected, this flatter diurnal patterning can lead to a higher overall cortisol release across the day [30,91]. 

The abnormal cortisol pattern is related to the circadian misalignment caused by the irregular waking hours required of shiftworkers. Furthermore, workers suffering shortened sleep, through working longer shifts or rotating between roster-types also demonstrate cortisol release impairments [90]. Animal models also provide evidence that increased exposure to artificial light, an acute consequence of shiftwork (Figure 1), is associated with disrupted cortisol secretion patterns [92]. Relevant to our model, the dysregulation of cortisol release in shiftworkers could impair their skeletal muscle health by favoring a negative protein balance. These relationships between sleep disruptions, increased cortisol release and progressive muscle wasting have been very recently implicated in the development of sarcopenia [93]. We propose that the circadian misalignment and sleep disruption incurred by shiftworkers could also impair skeletal muscle health, independent of age.

## 3. Potential Countermeasures to Preserve Skeletal Muscle Health in Shiftworkers

The current review hypothesizes that shiftwork, through altered light exposure, disruptions to sleep, and changes in food intake patterns may impair skeletal muscle health. These populations may therefore need evidence-based strategies to help promote and preserve this fundamental human tissue, essential for short-term physical capacity and long-term metabolic health. Identifying and implementing successful countermeasures will not only benefit the individual shiftworker, but also improve the health of this essential workforce, underpinning the essential services they provide communities.

There have been several reviews dedicated to improving shiftworker health (e.g., Wright Jr. et al. [1] and Kecklund and Axelsson [6]). Many of these reviews target the factors identified in Figure 1 and therefore, strategies targeting circadian adjustment through manipulating light exposure, improving sleep quantity and quality, and overall diet, could also benefit skeletal muscle health. Rather than revisit these strategies, this final section will focus on countermeasures that target two of the main drivers of skeletal muscle synthesis—protein intake and resistance training. To do so, we will draw on experimental studies in humans and rodents completed in conditions that approximate one or more aspects of shiftwork (e.g., altered sleep timing). 

Dietary protein is an established, potent stimulator of muscle protein synthesis [17]. Res et al. [94] reported that in humans, a 40-g mixed protein beverage drank 30 min prior to sleep significantly increased overnight muscle protein synthesis as well as the circulation of essential amino acids when compared to a control group. Although these findings were observed in participants permitted a full night’s sleep, they do highlight the potential protective impact of protein ingestion for the skeletal muscle health of shiftworkers. This possibility is further supported by recent findings using rodent models. In rats, 96 h of sleep deprivation significantly decreased testosterone levels and elevated corticosterone (the predominant glucocorticoid in rats) levels [38]. Supplementation by the essential amino acid Leucine did not protect against these hormonal changes; however, it was able to counteract the sleep deprivation-induced reduction in muscle fibre CSA in type IIb, but not in type IIa muscle fibres. Leucine supplementation also rescued the sleep deprivation-induced decrease in markers of muscle protein synthesis, but not the sleep deprivation-induced increase in markers of protein degradation [38]. Although no study has replicated these findings in humans, additional protein ingestion does represent a plausible and practical countermeasure to help shiftworkers to preserve their skeletal muscle. Research into the timing and dose of the protein required to optimise skeletal muscle health across a range of shiftworking conditions is a worthwhile focus for future investigations.

Resistance training is another primary stimulator of muscle protein synthesis [95]. Therefore, it may represent an effective countermeasure to the decrease in muscle protein synthesis and increase in muscle protein breakdown we have proposed for shiftworking populations (Figure 1). We are unaware of any studies that have explored the direct impact of resistance training on skeletal muscle health in shiftworkers or in human participants following a period of sleep deprivation or restriction. There are, however, some allied findings in rodents to prompt further investigation into this countermeasure. Monico-Netto et al. [39] tested rats’ responses to 96 h of sleep deprivation, eight-week resistance training, and 96 h sleep deprivation following the eight-week resistance training protocol. Resistance training alone increased muscle mass and CSA, while sleep deprivation alone significantly reduced muscle mass and CSA. When combined, resistance training attenuated the changes in muscle morphology and attenuated the reduction in circulating levels of anabolic hormones that were induced by sleep deprivation. In addition, resistance training was able to blunt, but not fully suppress, the sleep deprivation induced increase in corticosterone levels and protected against the increase of some, but not all, muscle protein degradation markers. These findings have yet to be replicated in human participants. The concurrent impact of chronic sleep restriction (and other sleep disruptions common to shiftwork) and resistance training is also yet to be explored, but the findings of Monico-Netto et al. [39] are promising and should prompt further research in humans and, in particular, shiftworking populations. 

For researchers and practitioners implementing resistance training programs in shiftworking populations, research using acute periods of total sleep deprivation and partial sleep restriction provide some insights that could be used to optimize training prescription. Reilly and Piercy [96] showed that participants found their sub-maximal bench press exercise at 3 a.m. was significantly more demanding (i.e., associated with a higher rating of perceived exertion) than when the same load was lifted at 3 p.m. The experimental design was not continued to examine a training effect, nor could the impact of the time of day be separated from the sleep deprivation incurred by keeping participants up until 3 a.m. However, given that perceived exertion is a key driver of self-selected work intensity, then shiftworkers training following impaired sleep may not be able to (a) lift as much load in their training and/or (b) progressively overload their training load sufficiently to optimize protein synthesis. Secondly, the increased exertion of training, especially if coupled with a lack of progression, could de-motivate shiftworkers to undertake resistance training. To counter these potential impairments, workplaces should consider the positive influence that exercising in pairs or teams may have on motivation and exertion [97,98], two drivers of physical activity and training that are impaired by sleep restriction [96,99]. Indeed, in a highly competitive team weight-lifting environment, Blumert et al. [100] found that maximal strength is not impaired by 24 h of complete sleep deprivation. We have also shown that firefighters working in teams will maintain their physical performance on important tasks without a decrease in motivation or perceived exertion [101]. Neither of these acute sleep restriction or deprivation environments are a true proxy for the chronic sleep disruptions commonplace for shiftworkers. However, researchers seeking to trial resistance training programs with shiftworking clients should strongly consider the value of exercising in groups or with partners to buffer the normal drop in motivation or perceived exertion associated with sleep deprivation and restriction.

## 4. Conclusions

The current review hypothesized that shiftwork may significantly impair skeletal muscle health through multiple physiological pathways resulting in a reduction of protein synthesis and an augmentation of protein degradation in the muscle. Our conceptual model explored relationships between typical shiftwork consequences; altered light exposure, sleep patterns, and food and beverage consumption, and drivers of skeletal muscle health—protein intake, resistance training, and hormone release. To date, no study has directly investigated the skeletal muscle health of shiftworkers. In the absence of direct evidence, we drew from some experimental findings that altered light, sleep, and eating patterns can directly influence skeletal muscle homeostasis. Emerging data from human and rodent laboratory experiments suggests that targeted protein ingestion and/or resistance training may be viable strategies to preserve skeletal muscle health for shiftworkers. Further research is required to test the potential relationships identified in our review, including the efficacy of the proposed interventions.
